# Proficiency testing of skin prick testers as part of a quality assurance system

**DOI:** 10.1186/s13601-016-0126-7

**Published:** 2016-09-21

**Authors:** Hans-Jørgen Malling, Pernille Allesen-Holm, Lisbeth Sys Karved, Lars K. Poulsen

**Affiliations:** Allergy Clinic, Copenhagen University Hospital Gentofte, Kildegårdsvej 28, 2900 Hellerup, Denmark

**Keywords:** Coefficient of regression, Coefficient of variation, Histamine skin prick test, Reproducibility, Quality assessment

## Abstract

**Background:**

Skin prick test is an important diagnostic procedure in clinical allergy but documentation of the quality is often missing.

**Methods:**

We describe a proficiency system to evaluate staff members in relation to the international recommended reproducibility in terms of coefficient of variation (CV < 40 %) and the linearity (coefficient of regression >0.85) based on blinded octuplicate histamine testing using histamine 3, 10, 30 and 100 mg/ml.

**Results:**

Fourteen trained allergy nurses participated in the proficiency testing. More than 95 % of the nurses, generated coefficient of variation less than 40 %, and for around 35 % of testers the CV were below 20 % based on wheal area. Regarding the linearity (coefficient of regression), only two nurses produced tests with a value below 0.85. On the contrary, 79 % of testers demonstrated a coefficient of regression >0.95. Depending on the gentleness of the prick procedure, the inter-nurse variability in wheal area varied more than twofold corresponding to a 10-doubling of histamine concentration. This would never have been detected without using a proficiency testing system.

**Conclusion:**

The described histamine testing provides an objective system for the evaluation of basic skin test quality assessment standards especially for documentation in scientific studies.

## Background

Skin prick test (SPT) is one of the diagnostic cornerstones in IgE-mediated allergic diseases [1]. It is widely accepted due to the safety, convenience and cost-effectiveness [2, 3]. It is, however, prone to variability that may fundamentally change the readout of the test. Standards for optimal performance of the SPT have been provided [2, 4, 5]. In the daily routine the SPT are normally performed by nurses or technicians. Based on the position as a “gold standard” in allergy diagnosis [3] it is important to bring the SPT proficiency up to a predefined minimal standard and to be able to document this. As quality assurance is becoming increasingly demanded, it is crucial also to bring a biological test into better alignment with other diagnostic tests where quality assurance is an integrated component of performing the test. A suggested proficiency testing protocol was presented in 2008 by The American Academy of Allergy, Asthma and Immunology (AAAAI) and the American College of Allergy, Asthma and Immunology (ACAAI) joint Update Practice Parameter [2]. In spite of that, a recent paper [6] calls for standards and consistency of the test due to a described gab between recommendations and daily practice. Especially in publication of clinical studies, proficiency testing data for SPT performance should be reported.

This study describes a quality assurance system to evaluate and to document the reproducibility of skin prick testers. Due to the extensiveness of this system, it is suggested to perform this test yearly or every second year.

## Methods

### Study population

All skin test were performed on only one individual (HJM) between 8 am and 4 pm over a period of 2 months with at least 1 day interval. The subject did not suffer from allergy or skin diseases and had not been treated with any substances interfering with the histamine induced skin reaction [5].

### Skin prick test

The testing was performed double-blind using bottles numbered 1–32 which included 8 bottles of four concentrations of histamine hydrochloride (3, 10, 30 and 100 mg/ml) in 50 % glycerol applied in random order. Each test session consisted of 32 individual SPTs with 8 repeats of each of the four histamine concentrations. Negative controls in duplicate were performed with saline. The tests were applied on the forearms spaced 3 cm apart by the “puncture method” using the same type of a 1 mm lancet (device applied at 90° angle with downward pressure) (7). Devices were used once and then discarded [7]. The wheal reactions were read after 15 min, outlined and transferred by tape to paper. The area in mm^2^ was measured by PC-based planimetry witch previously has shown a documented CV of 2–3 % [8]. Positive skin test was defined as a wheal area ≥7 mm^2^ [2, 4, 5]. The consistency in producing identical wheal areas was expressed as coefficient of variation (CV) and was calculated for the octuplicate tests for each histamine concentration and testing personal. The linearity (dose–response relationship) was calculated as correlation coefficient in a log area vs log histamine concentration based on all tests per concentration [5]. All nurses employed at the Allergy Clinic, Copenhagen University Hospital Gentofte were included in the proficiency testing. Some nurses were newly employed with limited experience but carefully trained whereas others had more than 20 years of experience.

## Results

Fourteen testers completed the proficiency study. The negative controls resulted for most operators in completely negative reactions and for all operators in wheal reactions less than 7 mm^2^. This “background” was not subtracted from the histamine reactions.

**Fig. 1:**
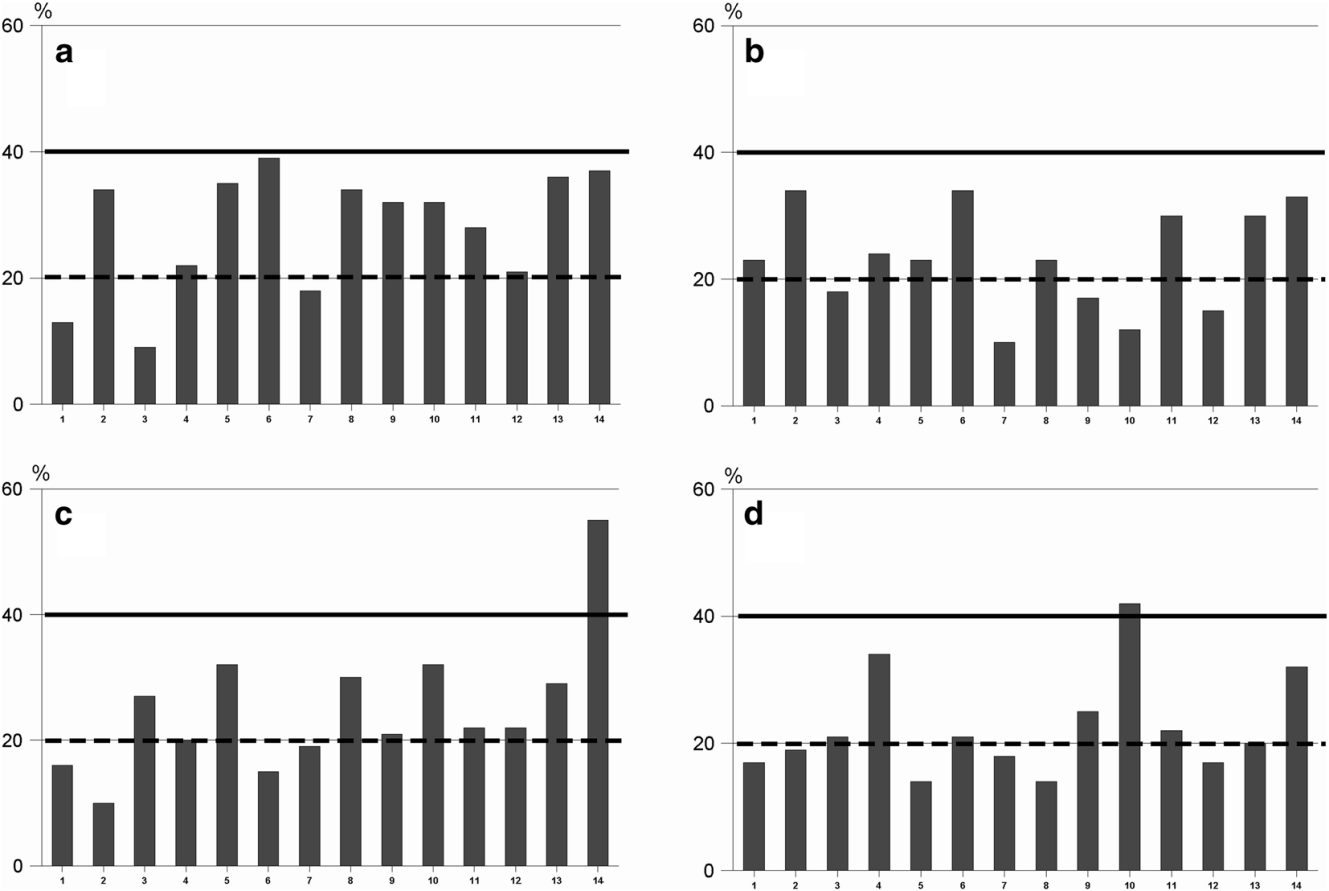
The coefficient of variation of 8-double histamine wheal areas. **a** depicts the results of 3 mg/ml; **b** 10 mg/ml; **c** 30 mg/ml and **d** 100 mg/ml histamine. The solid line indicates the upper limit (CV < 40 %) for acceptance (4,5) while the dotted line represents the CV of 20 % (highly qualified tester)

Figure 1 shows that the majority of operators were able to obtain a CV less than 40 % as 96.4 % of the tests came below this recommended maximum value. Around 35 % of operators produced a CV below 20 %. In order to accept the linearity in a log–log system the coefficient of regression must be >0.85. Two testers (14.3 %) failed to reach this goal. Positively, 79 % of testers demonstrated a coefficient of regression >0.95.

**Fig. 2:**
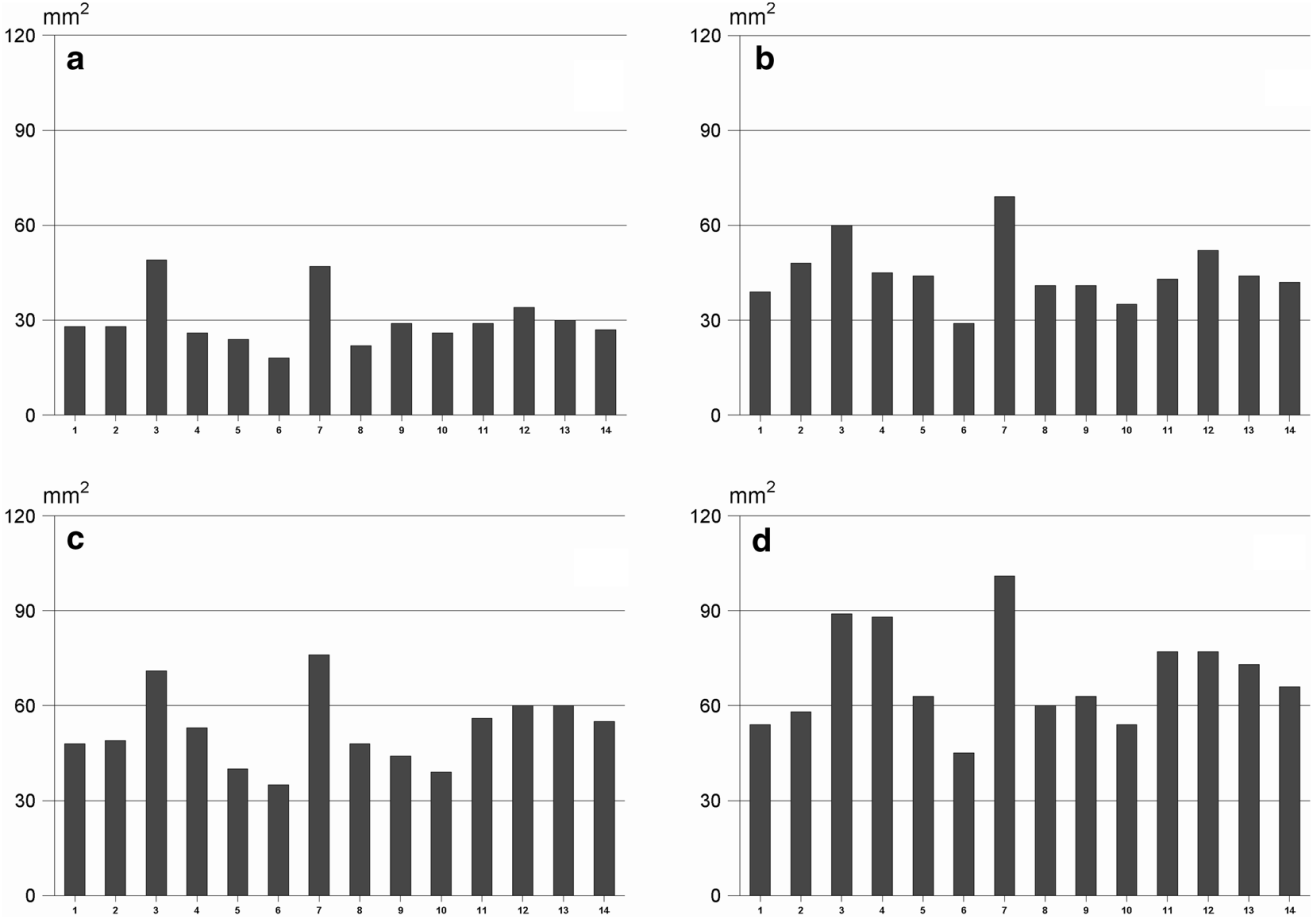
Mean wheal area (square millimetres) of the 8 repeats of each of the 4 different concentrations of histamine of the 14 nurses tested. Histamine concentrations as in Fig. 1

**Fig. 3:**
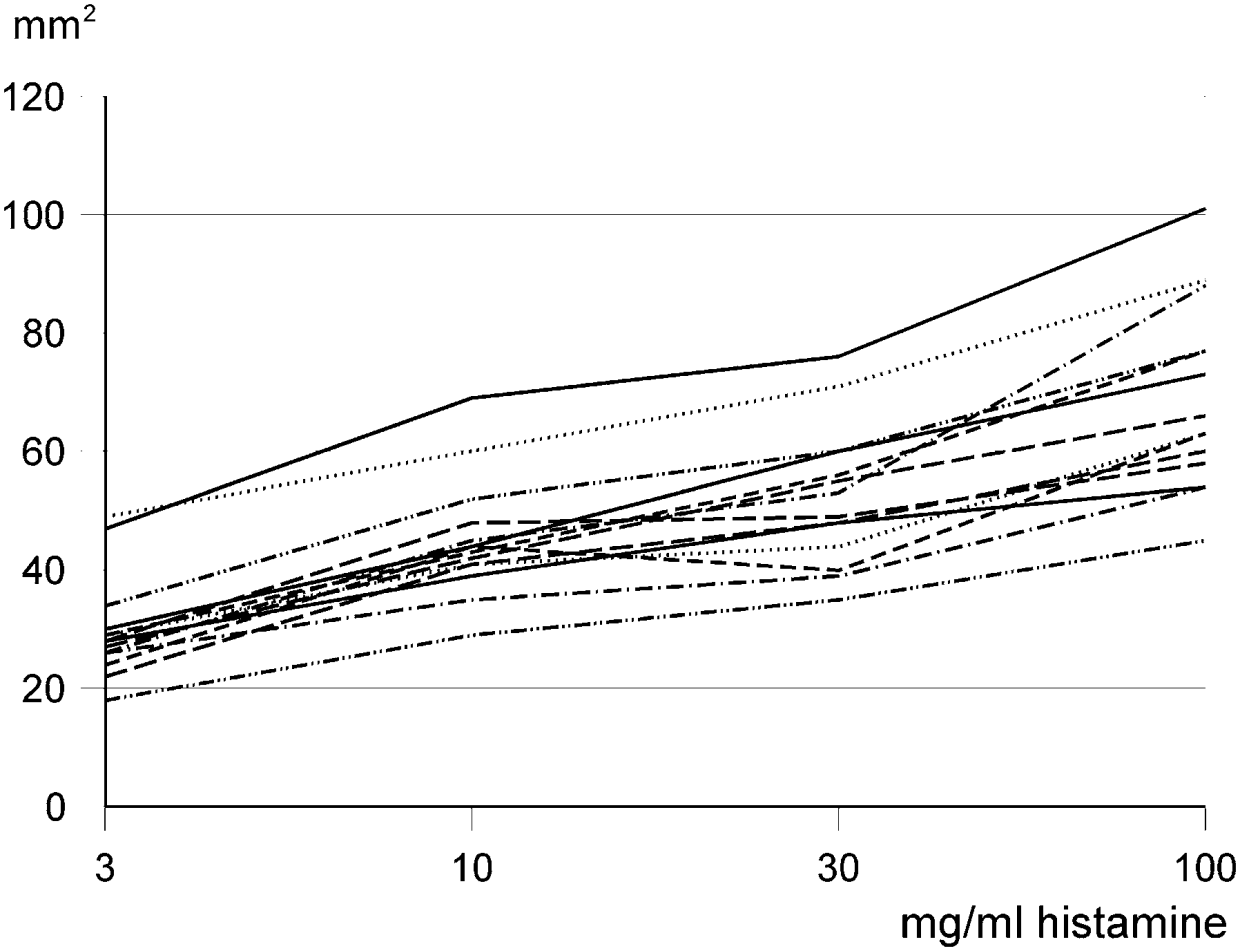
Dose-response curves of wheal area vs histamine concentration of all 14 tested operators. The median slope is 0.217 corresponding to almost a doubling of the wheal area by increasing the histamine concentration by a factor 10

The wheal area of the 4 different concentrations of histamine is shown in Fig. 2. The median area of histamine 3 mg/ml was 28 mm^2^, 41 mm^2^ for histamine 10 mg/ml, 49 mm^2^ for histamine 30 mg/ml and 63 mm^2^ for histamine 100 mg/ml. Some operators produced large skin reactions (like tester #3 and #7) with all concentrations tested, while other consequently produced small reactions (tester # 6). The difference between individual testers corresponded to more than a doubling of the area. The slope of the log–log dose response curves varied from 0.168 to 0.279 (median 0.217) equivalent to slightly less than a doubling of wheal area by 10-doubling the histamine concentration (Fig. 3).

## Discussion

In order to bring SPT into better alignment with areas of medicine where quality data are required, proficiency testing based on specific standards should be requested. Data from 2006 indicate a need as up to 90 % of practicing allergists did not assess the reproducibility of their testing staff’s skills [9]. The European standard for skin prick testing implies a coefficient of variation based on wheal diameter less than 20 % (40 % with wheal area) after histamine control applications and dose–response relationship calculated as correlation coefficient higher than 0.85 [5]. A coefficient of variation of less than 30 % (diameter) has been advised by the 2008 American Academy of Allergy, Asthma and Immunology and the American College of Allergy, Asthma and Immunology Joint Update Practice Parameter [2]. In the present study, the operators nicely fulfilled the requirements as regards reproducibility (CV %) and linearity (R^2^). In spite of minor deviations the increase in wheal area is constant over the range of histamine concentrations used (Fig. 3), but the operators were not able to produce inter-tester comparable wheal areas. The difference between different operators corresponds to at least a tenfold difference in histamine concentration. This substantial inter-tester variability does not seem to be related to variability in histamine responsiveness nor to the outlining of the wheal, but to the very performance of the SPT i.e., the gentleness of the prick procedure. The quality of the test was not convincingly related to the years of experience of the testers.

In daily clinical practice the skin response is either a “positive” or “negative” categorical assessment, but in scientific studies the results may often be interpreted quantitatively. In the scientific literature documentation of quality assurance standards being fulfilled is often missing [6]. Unless allergen sensitization is read as histamine equivalent reaction [10] it is important to produce wheal areas of a constant size and the force of the prick procedure seems to be crucial in this aspect. Producing small wheal reactions may imply that weakly sensitized patients may be missed due to a false negative skin test. To accurately interpret results of SPT in these settings, it is important to document a low-level inter-tester variability when performed by multiple operators [11]. The titrated histamine testing gives an easily assessable evaluation of the inter-tester and intra-tester reliability and quality in performing SPT.

Compared to the 2008 American Suggested Proficiency Testing and Quality Assurance Technique for SPT [2] the method suggested in this paper implies several advantages: (1) all tests are applied on only one individual and thereby ruling out intra-individual recipient differences as being the basis for observed differences between individual operators; (2) planimetry imply a more precise measurement of the wheal than using the mean diameter [8]; (3) the titrated test offers the possibility of evaluating the linearity (coefficient of correlation).

The histamine proficiency testing provides an objective system for making quality assurance evaluation without exposing allergic patients to unnecessary hazardous and unpleasant testing using allergen extracts. The results of histamine testing may not be directly transferable to testing with allergens, but it does give valid information how staff members performing SPT in reality meet basic quality assurance standards.

In centres not possessing equipment for planimetry, the mean diameter may be used, but we prefer planimetry due to a higher precision. For scientific purposes, SPT proficiency testing represents an objective standard to verify operator qualification and should be integrated in a centre’s standard quality assurance programme.

We are about to initiate a project focusing on self-training to be able to induce wheal areas within a pre-defined range (diameter 7 ± 1 mm; area 28–50 mm^2^) using histamine 10 mg/ml in order to be able to compare actual allergen induced wheal size between individual operators [12].

## Conclusion

Documenting the quality of skin prick testing is essential especially in scientific studies. We describe an objective proficiency system using histamine testing for documenting basic skin test quality standards. Histamine testing imply not running the risk of inducing severe allergic reaction as might be the case with allergens and by using only one test person we minimize other variables, that might confound the outcome. We suggest performing proficiency testing annually or biannually for quality assurance.

## Abbreviations

SPT: skin prick test
CV: coefficient of variation
R^2^: coefficient of regression
